# Ischemic Stroke Detection System with a Computer-Aided Diagnostic Ability Using an Unsupervised Feature Perception Enhancement Method

**DOI:** 10.1155/2014/947539

**Published:** 2014-12-09

**Authors:** Yeu-Sheng Tyan, Ming-Chi Wu, Chiun-Li Chin, Yu-Liang Kuo, Ming-Sian Lee, Hao-Yan Chang

**Affiliations:** ^1^School of Medicine, Chung Shan Medical University, No. 110, Section 1, Jianguo North Road, Taichung 40201, Taiwan; ^2^Department of Medical Imaging, Chung Shan Medical University Hospital, No. 110, Section 1, Jianguo North Road, Taichung 40201, Taiwan; ^3^School of Medical Imaging and Radiological Sciences, Chung Shan Medical University, No. 110, Section 1, Jianguo North Road, Taichung 40201, Taiwan; ^4^School of Medical Informatics, Chung Shan Medical University, No. 110, Section 1, Jianguo North Road, Taichung 40201, Taiwan

## Abstract

We propose an ischemic stroke detection system with a computer-aided diagnostic ability using a four-step unsupervised feature perception enhancement method. In the first step, known as preprocessing, we use a cubic curve contrast enhancement method to enhance image contrast. In the second step, we use a series of methods to extract the brain tissue image area identified during preprocessing. To detect abnormal regions in the brain images, we propose using an unsupervised region growing algorithm to segment the brain tissue area. The brain is centered on a horizontal line and the white matter of the brain's inner ring is split into eight regions. In the third step, we use a coinciding regional location method to find the hybrid area of locations where a stroke may have occurred in each cerebral hemisphere. Finally, we make corrections and mark the stroke area with red color. In the experiment, we tested the system on 90 computed tomography (CT) images from 26 patients, and, with the assistance of two radiologists, we proved that our proposed system has computer-aided diagnostic capabilities. Our results show an increased stroke diagnosis sensitivity of 83% in comparison to 31% when radiologists use conventional diagnostic images.

## 1. Introduction

Stroke, also known as a cerebral vascular accident (CVA), is the most common and the most threatening cerebrovascular condition and is one of the main factors contributing to the increase in global mortality. Statistics tell us that twenty-five to thirty million of the five billion people living around the world have suffered a stroke [1]. Stroke has ranked third among the ten leading causes of death in Taiwan for many years, with approximately 10,000 people in Taiwan dying from stroke every year. Mayer et al. [2] reported that the patients who have survived a stroke typically became impaired in their mobility and thus require the expenditure of a large amount of family, social, and medical resources. Stroke can be divided into two types: ischemic and hemorrhagic [3, 4], both of which are major causes of morbidity and mortality worldwide. In recent years, it has become clear that systemic inflammation may enhance atherogenesis [5, 6], which leads to stroke.

With respect to stroke risks, various computer-aided diagnosis (CAD) systems have been developed to assist physicians in the diagnosis and treatment of stroke patients [7–9]. These systems have made possible the detection of early CVA signs and have contributed to the improved diagnostic accuracy for acute strokes.

Current stroke detection devices such as computed tomography (CT) and magnetic resonance imaging (MRI) can help an experienced radiologist to determine whether or not a patient has suffered a stroke, but if a general radiologist makes an erroneous judgment, this may cause the patient to miss the best time for treatment [10]. Therefore, enhancing the quality of the diagnostic image is a critical way to help the physician make a proper diagnosis or image recognition.

Pižurica et al. [11] described a robust wavelet domain method for removing noise in medical images. While this method has low-complexity characteristics, both in its implementation and execution time, the processed image becomes blurred when using this method. It also requires higher time requirements, so this method is not ideal for use in stroke detection. Lin and Chin [12] proposed a new algorithm for the detection and compensation of backlight images which addresses the weaknesses of conventional backlight image processing methods such as oversaturation and lack of contrast. This method can produce high image contrast and is used in medical image contrast enhancement [13]. Ušinskas et al. [14] used 18 joint textural features to search for ischemic stroke patterns in CT slices, but the automated thresholding for each image, at the time of this study, was not complete.

Lin and Liu [15] proposed a neural network technology based on artificial intelligence which constructs a prediction model to detect recurrent stroke. Although this model is useful for discharged patients, certain difficulties remain in tracking and collecting relevant patient information. Lee et al. [16] proposed a method to find feature points that smoothens CT images, analyzes the edge of the brain tissue, and then applies a Gaussian-weighted distance model to obtain feature points to reconstruct a 3D human brain model. This method enables radiologists to find the likely stroke area quickly. However, the disadvantage of this method is that patients have to endure prolonged CT scanning that may delay critical medical treatment for acute stroke patients and can result in progression of the stroke. Rutczyńska et al. [17] proposed a method that uses regional growth in conjunction with Gaussian mixture models (GMM) to calculate the maximum expected value and then uses Bayes theorem to update the maximum chance of finding the stroke area. This method is only effective for large stroke region areas and fails to effectively detect areas of early stroke. Chawla et al. [18] developed an automated method to detect and classify lesions as being an acute infarct, chronic infarct, or hemorrhage at the slice level of noncontrast CT images. This method consists of three main steps: image enhancement, detection of midline symmetry, and classification of abnormal slices. First, a windowing operation is performed on the intensity distribution to enhance the region of interest. Then, domain knowledge about the anatomical structure of the skull and the brain is used to detect abnormalities both in a rotation- and translation-invariant manner. Finally, a two-level classification scheme is used to detect abnormalities using features derived in the intensity and the wavelet domains.

Regarding MRI applications and related literature, Kesavamurthy and SubhaRani [19] used a variety of different image analysis technologies to process brain tissue images obtained from MRI technology and also depicted accurately damaged areas of the brain tissue with a semiautomated technology that improved clinical diagnosis and treatment. In this paper, the author describes an edge detection method which uses a Canny edge detection algorithm to extract damaged areas of brain tissue in order to accurately distinguish the boundaries of the adjacent normal brain tissues and skull.

Zhang et al. [20] proposed that object boundary definition is an important task in brain image analysis. The brain structure can be used to improve the disease detection rate at earlier stages. Recently, a 3D active volume model (AVM) was proposed which incorporates both gradient and regional information to enhance robustness. However, the segmentation performance of this model depends on the position, size, and shape of the initialization, especially for data with complex texture. Mangla et al. [21] explored the edge regions of the human brain to observe and study the incidence of stroke and recognized two types of border zone infarcts: external (cortical) and internal (subcortical). Strokes occurring in the white matter are most commonly hemorrhagic. In contrast, ischemic strokes often occur in the gray matter. The author proposed a combination of several advanced techniques useful in identifying pathophysiologic processes.

From a survey of the literature, the threat and seriousness of ischemic stroke are widely recognized but, as yet, an advanced diagnostic technique for ischemic stroke has not been yet made available. Przelaskowski et al. [22] enhanced the overall contrast of brain images, but his method does not map the stroke position with high accuracy. Instead, the stroke area can be highlighted after processing. But the processing itself may blur out the stroke area and increase the diagnostic time, leading to delays in treatment. Some studies have used neural networks with high accuracy, but they have not yet established that they can successfully identify a stroke area in the improved image. As a result, they use semiautomatic methods to determine the threshold values of different images [23]. There remain some problems in identifying the brain stroke area. First, high resolution equipment is expensive. Secondly, the diversity of human brain tissue increases the difficulty of diagnosis. Lastly, the presence of an old brain stroke area can also make it difficult to distinguish a new stroke area from the old one. These problems often lead to delay in treatment and cause anxiety and regret for families. To solve these problems, we propose an unsupervised brain stroke detection system that can accurately and quickly highlight the affected stroke area.

## 2. Material and Methods

**Figure 1:**
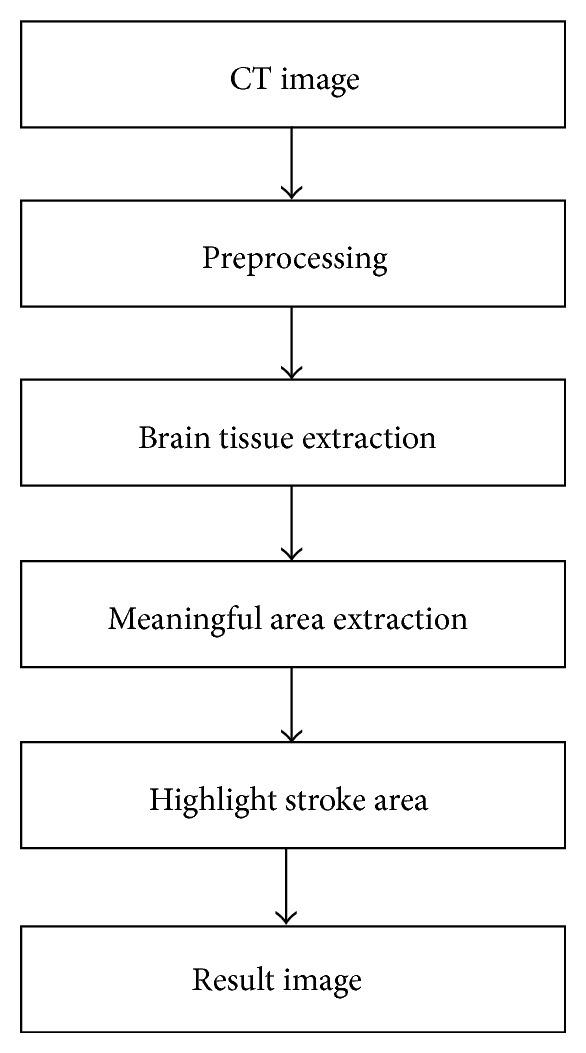
System flowchart for ischemic stroke detection.

Figure 1 shows our proposed detection flowchart for ischemic stroke. It is divided into four processing steps: preprocessing, brain tissue extraction, meaningful area extraction, and highlight stroke area.

### 2.1. Preprocessing

**Figure 2:**
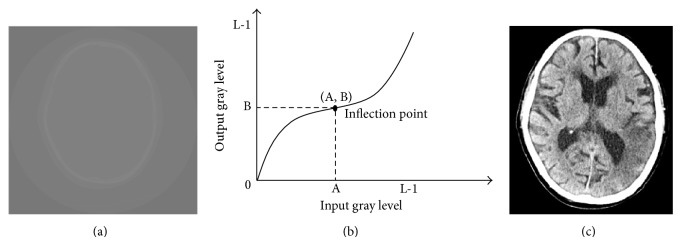
(a) The original brain image, (b) the cubic curve of the contrast enhancement method, and (c) the resulting image obtained after performing contrast enhancement.

In a low-contrast conventional brain CT image, as shown in Figure 2(a), the image contrast is insufficient. Using our method, we first input a brain CT image like the one shown in Figure 2(a) to our system. To perform preprocessing, we use the cubic curve contrast enhancement method [13, 24] to improve the image contrast and make its texture clearer.  Figure 2(b) shows the cubic function of the contrast enhancement curve. From this cubic curve, we can determine the coordinates (*A*, *B*) of the inflection point and then enhance the image contrast.

Equation (1) shows the cubic curve equation, where *x* represents the pixel value of the original image and *y* represents the pixel value of the intensified image. Because the curve passes through the origin (0, 0), *d* can be omitted

(1) $$\begin{array}{c} y=f\left(x\right)=ax^{3}+bx^{2}+cx+d. \end{array}$$

Before calculating the correlated coefficients, *a*, *b*, and *c*, we use (2) to determine the *x* coordinate, *A*, of the inflection point

(2) $$\begin{array}{c} A=\underset{x\in I}{\min}\left\{x\right\}+0.7\left(\underset{x\in I}{\max}\left\{x\right\}-\underset{x\in I}{\min}\left\{x\right\}\right). \end{array}$$

In (2), *A* represents the *x* coordinate of the inflection point, *I* represents the image, and *x* is any one of the pixel values in the image. Then, based on (3), we can calculate the contrast-enhanced cubic curve

(3) $$\begin{array}{c} c=1-a\times {\left(255\right)}^{2}-b\times 255,\\ b^{3}=3\times a-{\left(255\right)}^{2}\times 3a^{2}-255\times 3\times a\times b,\\ a=\frac{1}{{\left(255\right)}^{2}-3\times 255\times A+3\times A^{2}}. \end{array}$$

Figure 2(c) shows the resulting image after performing image contrast enhancement. From this figure, we can clearly see every tissue and region in the brain CT image.

### 2.2. Brain Tissue Extraction

**Figure 3:**
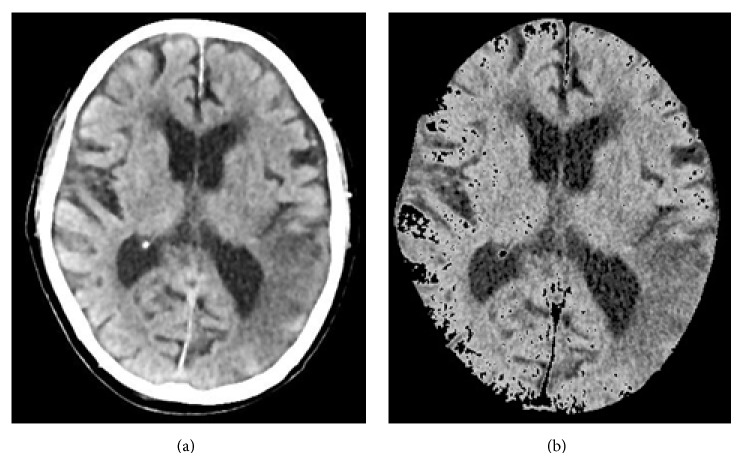
(a) Image after performing anisotropic diffusion and (b) the brain tissue image.

After visualization, the next step is to identify and remove those parts of the image that are nonbrain tissue, such as the skull, nearby areas, and other areas not affected by the stroke. First, we take advantage of the obvious differences in values between the skull and the brain tissue and use the Otsu method to calculate an optimal threshold value to obtain the most appropriate threshold. We then remove obvious skull images and noise. Next, we use an anisotropic filter for blurring processing, which enables us to reduce noise and increase the brightness of regional differences in the image, according to (4) [25]. An example of the image we obtain by this method is shown in Figure 3(a)

(4) $$\begin{array}{c} I\left(x,y\right)=\frac{\sum_{i=-1}^{+1}\sum_{j=-1}^{+1}I\left(x+i,y+j\right)G\left(x,y\right)}{\sum_{i=-1}^{+1}\sum_{j=-1}^{+1}G\left(x,y\right)},\\ G\left(x,y\right)=\exp\left(-\frac{{\left|\sqrt{G_{x}^{2}+G_{y}^{2}}\right|}^{2}}{2k^{2}}\right),\\ \left(G_{x},G_{y}\right)=\left(\frac{\partial I\left(x,y\right)}{\partial x},\frac{\partial I\left(x,y\right)}{\partial y}\right). \end{array}$$

*I*(*x*, *y*) represents an original image, *G*(*x*, *y*) represents the Gaussian kernel function, and *k* is the variance of the Gaussian mask. These are chosen on the basis of the magnitude of the gradient computed in a 3 × 3 window.

Finally, we use the morphology erosion and expansion method to remove any remaining subtle skull images to obtain complete brain tissue images and to proceed with image segmentation. The results are shown in Figure 3(b).

### 2.3. Meaningful Area Extraction

In this step, we distinguish the gray-white matter interface, which is the most common region for a stroke to occur. To do so, we use brain area segmentation and partition techniques.

#### 2.3.1. Brain Area Segmentation

**Figure 4:**
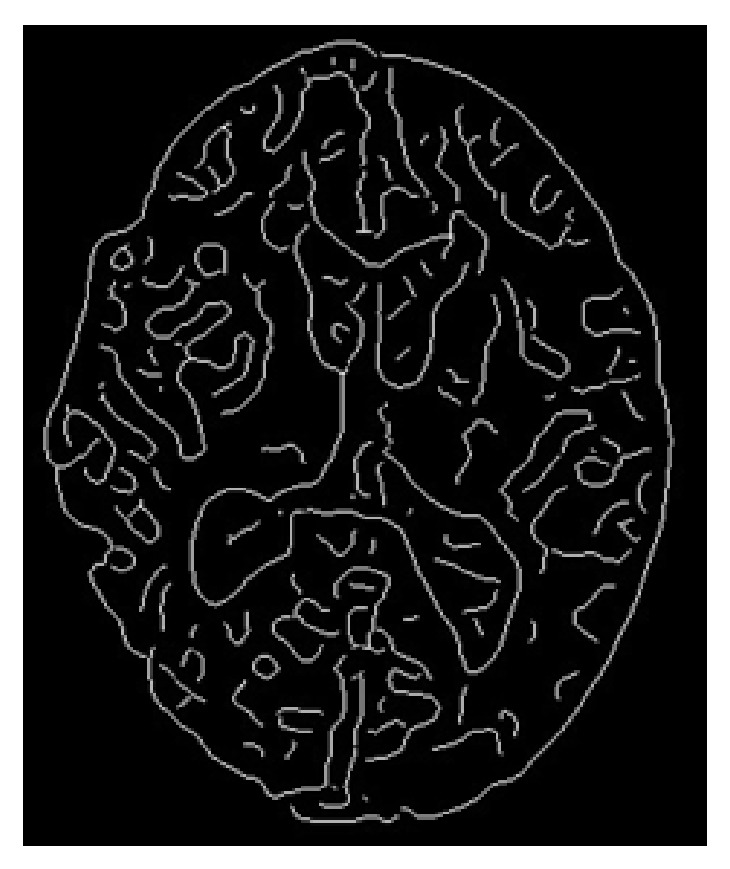
Edge of brain tissue image.

Professional radiologists know that the CT value of the brain tissue edge around a stroke area slightly differs from that of normal brain tissue. These differences are not easily detected for an inexperienced radiologist. To identify these slight differences, we use edge detection technologies and an unsupervised region growing algorithm (URGA) to make the differences more obvious. In edge detection, there is a qualitative change in the CT stroke image, so we want to identify the edge of the stroke area. We obtain image edge information by using the Canny edge detector to determine the optimal edge detection algorithm. This is an optimal edge detector that achieves good detection, good localization, and minimal response time, making it our method of choice for finding the edge of brain tissue. Our results are shown in Figure 4. Next, we propose the use of an URGA to segment the image as follows.

**Step 1 .**

Obtain an edge map using *I*_*e*_ = *I* ⊗ *G*, where *G* is a Gaussian filter, *I* is an original image, and ⊗ represents convolution.

**Step 2 .**

Calculate the histogram *H*(*i*) with the original image *I*_*e*_, in which the counted points are those nearby each edge point in the edge map *I*_*e*_.

**Step 3 .**

Denoise in the *H*(*i*) using a mean filter, where *H*(*i*) is the histogram obtained in Step 2.

**Step 4 .**

Obtain the peak value *H*(*i*) and set *i* as a seed. The condition of obtaining *H*(*i*) is as follow.

If ∑_*i*=4_^*L*−4^∑_*j*=0_^3^sign⁡(*H*(*i* + *j* + 1) − *H*(*i* + *j*)) = −4 and ∑_*i*=4_^*L*−4^∑_*j*=0_^3^sign⁡(*H*(*i* − *j*) − *H*(*i* − *j* − 1)) = 4, where ∀*i* ∈ *R*.

**Step 5 .**

Call the region_growing_algorithm (*i*, *I*), where ((*I*(*x*, *y*) − *T*)>(*μ* − *I*(*x*, *y*))).

**Step 6 .**

Repeat Steps 1 to 5 until *P*(*i*) is empty, and return the segmented regions on the brain tissue image *I*.

**Figure 5:**
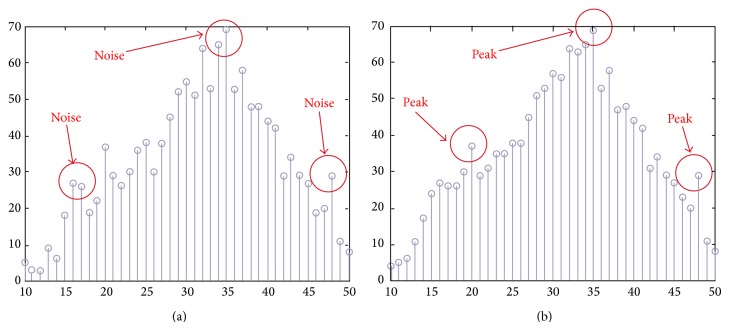
(a) Histogram obtained after performing URGA Step 2, with noise values framed in circles, and (b) the histogram after performing the mean filter.

**Figure 6:**
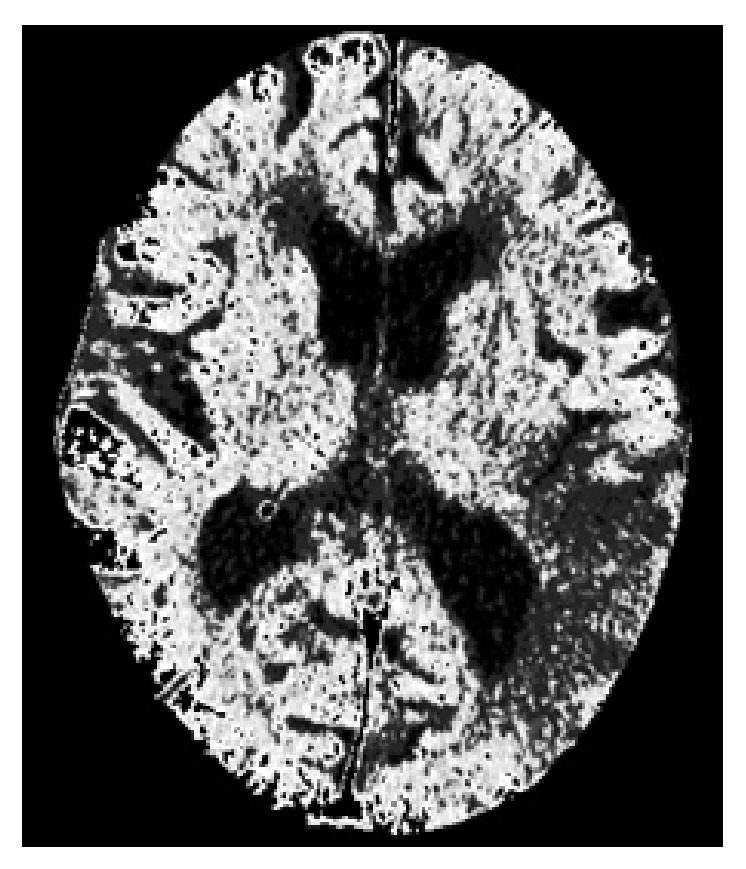
Image obtained after executing the URGA.

The aim of the URGA is to improve the traditional region growing algorithm which cannot automatically provide seed values. These seed values are extracted around the edge but not in the cerebrospinal fluid (CSF) area.  Figure 5(a) shows the histogram obtained after performing Step 2 in the URGA. In this figure, the position of the red arrows indicates the noise positions.  Figure 5(b) shows the histogram after performing the mean filter, where the noises have been removed and the red arrows indicate the peak positions. We regard these peaks as seed values. After obtaining the seed values, we continue with the region growing until the original image is completely segmented.  Figure 6 shows the resulting image after executing URGA.

#### 2.3.2. Brain Area Partition

After brain area segmentation, the next step, as reported by the radiologist consulted in this study, is for doctors to identify suspected stroke regions by comparing differences between the left and right side brain tissues. This knowledge should then be entered into our proposed system. To do so, we first use an image projection method to determine the maximum enclosing rectangle measurements in the brain tissue images, and, then using this rectangle as a basis, we use the center of the rectangle's length and width as the center coordinates of the brain tissue, thereby partitioning the brain tissue into four parts. We then distinguish the gray-white matter interface. Gray matter regions of normal brains are located in the periphery of the brain tissue and are brighter due to their higher cell density. In contrast, white matter is darker. So in our study we segment [21] the gray and white matter using a technique known by general radiologists, to generate an elliptic curve to determine the boundary between the gray and white matter.

**Figure 7:**
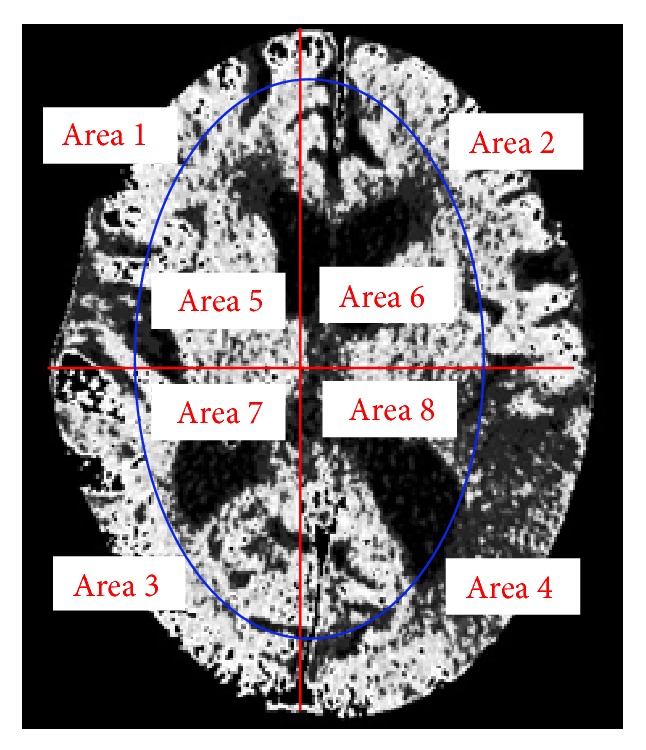
Image showing region partitions.

The center point of the internal and external ellipse is the same, but the length of the minor axis of the external rectangle is proportionally 2/3. Finally, we partition the brain tissue images into eight regions, as shown in Figure 7.

### 2.4. Highlight the Stroke Area

Physicians tell us that stroke occurs mostly in either the left or right side of the brain. Medical statistics show that the probability of stroke occurring in both the left and right sides of the brain is under 20%. The average brightness in the stroke area is less than the surrounding areas [26]. Therefore, we calculate the brightness values of all areas for comparison and then identify the positions and color and modify the image to make the possible stroke areas more obvious. By doing so, we can assist physicians to better observe and identify stroke areas and improve diagnostic accuracy.

#### 2.4.1. Coinciding Regional Locations

After partitioning the brain tissue images into eight regions, we next calculate the average brightness for each of the eight partitioned regions. Based on the knowledge that most strokes occur in one side of the brain only, we determine which area of the left or right brain is most likely to have experienced a stroke. To do so, we compare the calculated average brightness values for these eight regions. Finally, to identify regional locations for a stroke, we determine which areas have smaller brightness values by comparing the corresponding left and right side areas of the brain.

After identifying four areas with smaller average brightness values, we divide them into three cases, as shown in Table 1, according to their different distribution positions. In the first case, the four areas with smaller average brightness values are all on the same side. In this case, we can then determine the presence of a possible stroke area in either the left or right brain tissue from the smaller average brightness values that are identical. The second case has three areas with smaller average brightness values on the same side and one area with a smaller average brightness value on the other side. In this case, a judgment must be made.

**Figure 8:**
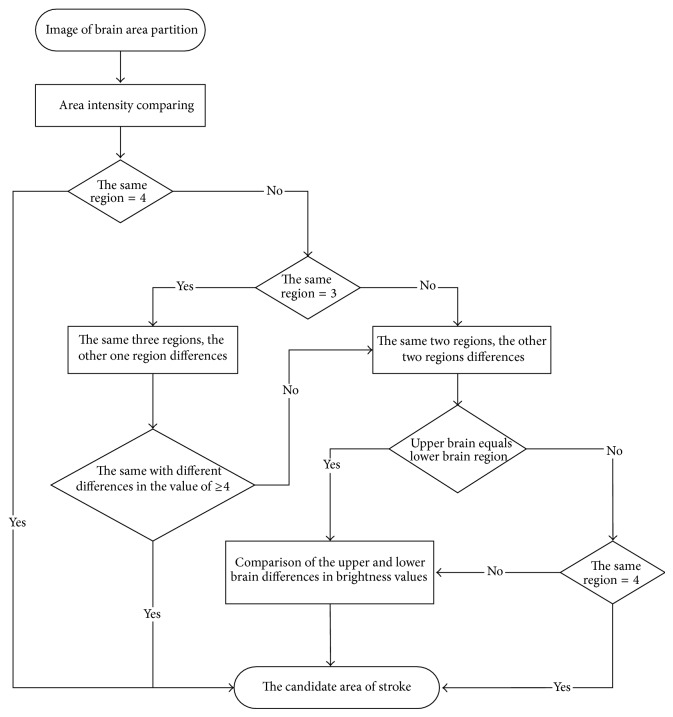
The flowchart of coinciding regional location.

The difference in brightness that can be distinguished by the naked eyes is 20 grayscales, which is equivalent to a brightness value of 4 [27]. Therefore, we set the differences of average brightness values to 4 in our study. Next, we calculate and compare the differences between areas having the same and different smaller average brightness values. If the difference in one area's brightness from that of the others is less than 4, we merge this area with the other three areas so that all four are designated as having smaller average brightness values in the left or right area. If the difference of one area's brightness is greater than or equal to 4, then we designate this area as being dissimilar to the other three areas on the same side. In the third scenario, areas can be further divided into two different distributions, wherein if two areas have similar smaller average brightness values and two other areas also have similar smaller average brightness values, then we must determine if the two area pairs with similar smaller brightness values are on the same side of the brain. If both pairs are on the same side of the brain, then we need to calculate the differences in the brightness values between the two. Having determined the location of the pair with the larger difference, we then merge the pair with the smaller difference with the pair with the larger difference so that the positions of these four areas coincide. This enables us to determine possible stroke areas. For the upper and lower parts of the brain tissue that are not on the same side, we calculate the differences in brightness between the left and right half-brain tissue areas for the upper and lower brain. Once we have determined the areas with smaller average brightness values that have the larger differences, we merge the small difference areas and then merge the large difference areas. We do this repeatedly for both the upper and lower brain areas, until the four regional positions coincide. The entire judgment flowchart is shown in Figure 8.

#### 2.4.2. Advanced Correction

From the position coincidence results, we then know whether the stroke occurred in the left or the right side of the brain. However, the system must further identify the stroke areas. In discussion with physicians, we learned that CT values between 30 and 36 indicate possible stroke regions. We are also informed that a stroke is more likely to occur in a dark area of the CT image than in the general brain tissue areas shown by the CT. So, we use 30% of the front of the brain tissue and temporarily assign CT values in this tissue as possible stroke area CT values. Next, we compare the colored images with the original images. If the CT value in the original lies within the 30% stroke area value, we mark it in red. If a value is not in this range, we restore the colored area to the original image value. Using this technique, physicians can better determine stroke areas and increase their diagnostic accuracy.

## 3. Experimental Results

For CT images, we used the Digital Imaging and Communications in Medicine (DICOM) format. We used an image resolution of 512 × 512, 4-bit color to store the metainformation and 12-bit color to view CT images at the 12-bit grayscale level. One pixel is equivalent to 0.2 mm.

In this experiment, we selected 90 CT and MRI images from 26 stroke patients, each with a stroke size in the range of 25 to 30 mm^2^. In our study design, one MRI image was selected by a radiologist. While ischemic stroke areas are obvious in MRI images, MRI testing is expensive and requires a lot of time. In general, people visit a doctor when concerned about possible symptoms of stroke. They do not typically take the initiative to undergo CT or MRI testing. Therefore, we do not have images from nonstroke patients. However, we did use MRI images to validate the accuracy of our proposed system. Next, we divided the experiment into three topics for exploration. First, we compared our proposed method with an existing method, proposed by Przelaskowski et al. [22]. Next, two radiologists tested our proposed system with respect to its potential for use as a computer-aided diagnostic technique. Finally, we used a statistical measure and an empirical discrepancy error measure known as object-level consistency error (OCE) [28], to evaluate our proposed system.

**Figure 9:**
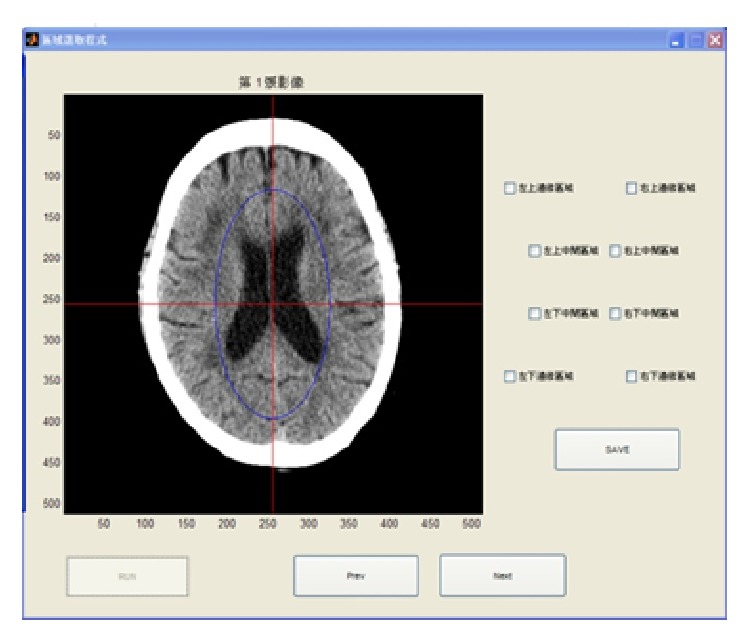
User interface for recording radiologists' assessment based on the original image (Test 1).

**Figure 10:**
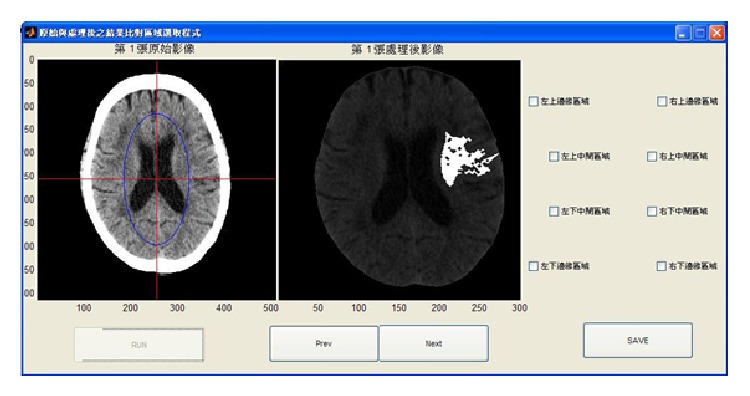
User interface for recording radiologists' decision after seeing the image results of our proposed system (Test 2).

Next, we designed two user programs for use by the radiologists in our study, the interfaces of which are shown in Figures 9 and 10. The aim of the first program is to enable radiologists, based on their professional experience, to select a brain stroke area from the four (or eight) areas drawn in our image. The program then saves this result to use during a follow-up assessment. The second program also allows radiologists to select the brain stroke area, but this time the radiologist also refers to the results from our proposed method. In this way, we tested whether or not the new information would change the judgment of the radiologists. Finally, we compare two classes of stroke patient brain images: the CT and the MRI.

**Figure 11:**
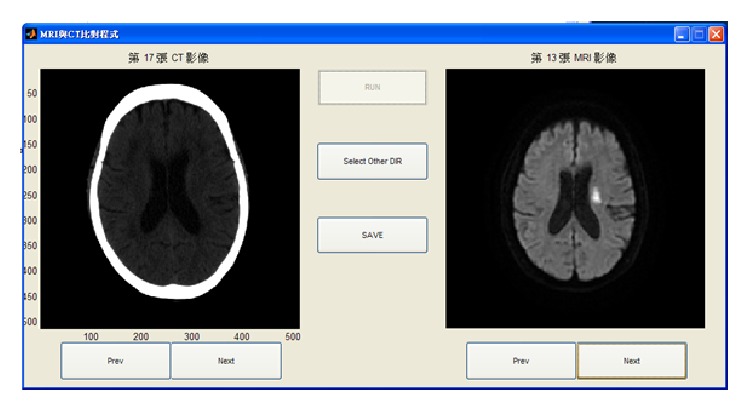
Typical user interface for obtaining an assessment by a professional radiologist. The left side shows a stroke patient CT image and the right side shows the corresponding MRI image of the same patient.

The objective of the third program, whose interface is shown in Figure 11, is to allow radiologists to manually determine the corresponding relationship between the CT and MRI images. Przelaskowski et al. [22] used a thresholding selection method to extract a brain tissue image using a dyadic wavelet transform, whose transform kernel is a biorthogonal filter bank with 5/3 taps of low pass filters, to enhance image contrast and remove noise. The resulting image is then converted to the spatial domain so the brain stroke area can be highlighted in the resulting image. We used the program provided by Przelaskowski to test a brain CT image obtained by the hospital of our school and compared the results of our proposed method with those obtained by the Przelaskowski method. The two participating radiologists also used this alternate program. They reported that the program did not easily facilitate the determination of the brain position of the stroke. The comparison results are shown in the middle column of Tables 2 and 3.

Table 2 shows brain imaging test results from four different stroke patients. In the left column is original image, in the middle is the image result from using wavelet-based processing methods for improved acute stroke detection, and in the right column are the image results using our proposed method. These results show that the use of edge detection and region growing for segmentation and colored marking enables the clear identification of the stroke area.

Table 3 shows the detection examples where our proposed system failed. After careful observation and statistical analysis, we found two reasons for these failures. The first was due to brain structure and the other had an old stoke area on one side of the brain, causing lower brightness values unrelated to the new stroke.

The locations of stroke can be clearly seen in MRI images, as shown in Figure 11. To validate the accuracy of our proposed method, two radiologists tested our proposed method in two experiments: Test 1 (Figure 9) and Test 2 (Figure 10). The results show whether the radiologists changed their opinions after seeing the image results from our proposed system. The radiologists also stated their belief that the number of divided regions in the brain CT image would affect their assessments. As a result, we assigned two different total numbers of regions. In one image, the brain is divided into our proposed eight regions. The other image is divided into four regions, at the suggestion of the radiologists. As reported by the radiologists, brain strokes typically cross two regions. Hence, we integrated the eight regions in Figure 9 into four regions.  Table 4 shows the results, in which there were two success rates between Tests 1 and 2 and one changed rate between Tests 1 and 2. We calculated the first success rate by dividing the number of images, in which the brain stroke area from the original image was correctly identified by the number of all the images. At this point, the two radiologists had not referenced the image result from our proposed system, and the success rate of radiologists 1 and 2 were 47% and 51% in images with four brain regions, respectively. The success rates of radiologists 1 and 2 were 33% and 40% in the images with eight brain regions, respectively. Next, we calculated the second success rate by dividing the number of images, in which the brain stroke area was correctly identified after seeing the image result from our proposed system, by the number of all the images. The success rates of radiologists 1 and 2 were 65% and 71% for images with four brain regions, respectively. The success rates of radiologists 1 and 2 were 60% and 64% for images with eight brain regions, respectively. Finally, the change rate is the percentage of different identification results between assessments based on seeing the original image only, and those made after also seeing the image results from our proposed system. The change rates of radiologists 1 and 2 were 57% and 63% for images with four brain regions, respectively. The change rates of radiologists 1 and 2 were 55% and 60% for images with eight brain regions, respectively.

The successful recognition rates of our proposed system for images having four and eight brain regions were 90% and 83%, respectively. From Table 4 results, we can observe three phenomena. Firstly, radiologists confirm that it is not easy to identify stroke areas in the brain from the original images. Secondly, the ratio of a radiologist successfully determining stroke areas increased. Finally, we see from the results that our proposed system effectively enhances the radiologists' ability to determine areas of stroke.

In medical diagnostic procedures, experimental accuracy must be rigorously evaluated prior to its acceptance. Therefore, in addition to the above results, we performed the following four statistical evaluations commonly applied in medical imaging. These include the TPN (true positive number), the TNN (true negative number), the FNN (false negative number), and the FPN (false positive number). We also used five performance factors to compare and validate the results generated by our two radiologists. These include specificity, precision, detection rate (DR), false alarm rate (FAR), and the correction classification rate (CR), calculated using (5), where DR is sensitivity/recall and CR is accuracy

(5) $$\begin{array}{c} \text{Specificity}=\frac{\text{TNN}}{\text{TNN}+\text{FPN}},\\ \text{Precision}=\frac{\text{TPN}}{\text{TPN}+\text{FPN}},\\ \text{DR}=\frac{\text{TPN}}{\text{TPN}+\text{FNN}},\\ \text{FAR}=\frac{\text{FPN}}{\text{TNN}+\text{FNN}},\\ \text{CR}=\frac{\text{TPN}+\text{TNN}}{\text{TPN}+\text{FNN}+\text{TNN}+\text{FPN}}. \end{array}$$

From the results shown in Table 5, we understand that our proposed system rates higher than 81% in DR, CR, specificity, and precision. However, the FAR value is too high.

Finally, we used the OCE to determine the evaluation error for the segmentation algorithm resolution at the object level. This method is superior for determining previous errors and can correctly identify whether there are an excessive number or too few split cases. It is also more sensitive than other methods in splitting the difference between results. The OCE results are less refined than natural scenes in which there is a high level of difficulty in increased task recognition. The results, shown in Table 6, show the global consistency error (GCE) and the local consistency error (OCE) [28].

Our proposed system, as presented in this paper, was developed and tested using MATLAB on Windows 8 computer with an Intel core i5-4200H 2.8 GHz processor and 8 GB of RAM and a method processing time of 1 s.

## 4. Conclusions

Ischemic stroke is the partial blockage of blood vessels in the brain that subsequently leads to brain tissue damage and can cause further brain damage,necrosis, and even death. This paper proposes an ischemic stroke detection system using a computer-aided diagnostic ability based on an unsupervised feature perception enhancement methodfor detecting ischemic stroke areas in brain CT images. This method may help radiologists to effectively diagnose stroke areas in a short time, while also reducing error rates. In addition, our proposed computer-aided diagnosis system was tested by two radiologists. The system mainly uses an unsupervised region growing algorithm to segment CT images into important areas and then uses a coinciding regional location method to highlight the stroke areas. Based on our experimental results from testing real CT images, the sensitivity of stroke diagnosis by radiologists increased to 83% from 31% when using conventional detection images.

## Figures and Tables

**Table 1:** Results of coinciding regional locations for different cases.

	Example 1	Example 2	Example 3
Original image	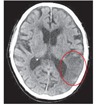	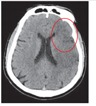	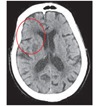

Step one	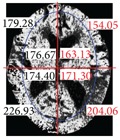	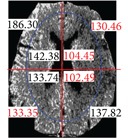	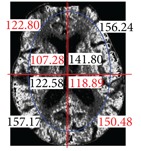

Step two		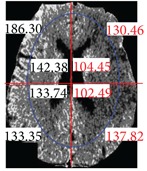	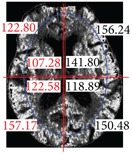

**Table 2:** Images from successful testing (left-to-right): original image, wavelet-based processing method for improved acute stroke detection, and increased visual perception brain stroke detection system.

Original image	Wavelet-based processing methods for improving acute stroke detection [22]	Our proposed method
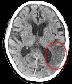	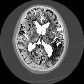	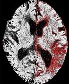

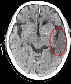	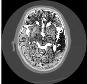	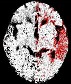

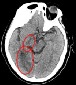	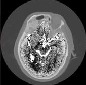	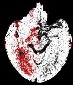

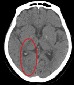	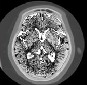	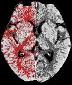

**Table 3:** Images in failed testing (left-to-right): original image, wavelet-based processing methods for improved acute stroke detection, and increased visual perception brain stroke detection system.

Original image	Wavelet-based processing methods for improving acute stroke detection [22]	Our proposed method
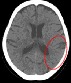	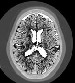	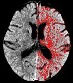

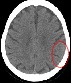	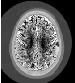	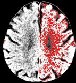

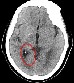	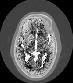	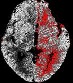

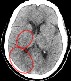	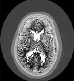	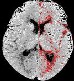

**Table 4:** Effectiveness of acute ischemic stroke detection in images with four brain areas.

	Radiologist 1		Radiologist 2	
	Four regions	Eight regions	Four regions	Eight regions
Successful rate of Test 1	47%	33%	51%	40%
Successful rate of Test 2	65%	60%	71%	64%
Change rate between Test 1 and Test 2	57%	55%	63%	60%

**Table 5:** Evaluation results of our proposed system in DR, FAR, and CR.

TPN	FPN	TNN	FNN	DR	FAR	CR	Specificity	Precision
51	6	26	7	87.93%	18.75%	85.55%	81.25%	89.47%

**Table 6:** CE error detection results of our proposed system.

	LCE	GCE	OCE
Procedure rate	0.1011	0.1509	0.8678
